# Supplementary material for: Anakinra for the Treatment of Hemophagocytic Lymphohistiocytosis: 21 Cases
Source: J Clin Med. 2022 Sep 30;11(19):5799. doi: 10.3390/jcm11195799 (PMC9572845; doi:10.3390/jcm11195799)
Supplement: Supplementary file 1 [file jcm-11-05799-s001.zip › jcm-1932222-supplementary.pdf]

Supplementary Data S1: Case description

Patient 1:

A 38-year-old man was diagnosed with disseminated tuberculosis with bone marrow involvement (PCR BK positive in the marrow) and concomitant Covid-19 infection. He had fever, splenomegaly, and pancytopenia with hyperferritinemia at 86000ug/l. The diagnosis of sHLH was made three days after anti-tuberculosis quadritherapy initiation. Anakinra (ANA) 100 mg/d (monotherapy) was started with a favourable evolution of the hyperinflammation, allowing its discontinuation after 13 days. After one month of anti-tuberculosis treatment, the patient had an immune reconstitution inflammatory syndrome (IRIS), without HLH recurrence; he was treated thus with corticosteroids alone, with a favourable outcome.

Patient 2:

A 33-year-old man was diagnosed with HIV infection (at AIDS stage C3) complicated by miliary tuberculosis, associated with EBV and CMV viral replications. Anti-tuberculosis treatment was initiated, complicated at 11 days by an IRIS requiring a short corticosteroid course. Anti-HIV therapy was started, after one month of anti-tuberculosis treatment, but was complicated by a new episode of IRIS with features of sHLH. Corticosteroids were resumed therapy and, due to an insufficient response, anakinra (at a dose of 100 mg/d) was combined with corticosteroids after two days. Anakinra dose was rapidly increased to 200 mg/d due to persistent fever peaks. The evolution was progressively favourable; anakinra and corticosteroids weaned after two months.

Patient 3:

A 74-year-old woman with a history of Crohn's disease, treated by azathioprine for several years, was admitted to hospital for prolonged fever associated with increased acute phase reactants. Azathioprine was discontinued without any clinical change. A PET scan didn’t show any pathological 18F-FDG uptake, osteomedullar biopsy did not identify any infiltration and the digestive endoscopies were normal. The viral PCR (EBV, CMV, HHV6, parvovirus B19) were negative. sHLH was diagnosed and corticosteroids were started at a dose of 2 mg/kg/d, with initial improvement. However, a recurrence of sHLH was observed after 15 days. The patient received one injection of etoposide (150 mg/m2), which allowed a transient resolution of clinical symptoms (during 7 days). IVIG treatment was ineffective and anakinra was started, in combination with corticosteroids (2 mg/kg/d), without any clinical or biological efficacy. The HLH-2004 protocol (etoposide, dexamethasone, CSA) was subsequently started with good efficacy and continued (weekly etoposide infusions). After an initial partial control of the disease, the evolution was rapidly unfavourable and the patient died five months after the beginning of the treatment, without any infectious or malignant aetiology having been demonstrated in this immunosuppressed patient.

Patient 4:

A 66-year-old man had persistent fever and sHLH in the setting of a myelodysplastic syndrome (excess blasts secondary to radio-chemotherapy for lung adenocarcinoma). EBV replication

was identified (3.2 log); the PET-scan revealed no significant 18F-FDG uptake. Fever persisted despite treatment with piperacillin/tazobactam, which was then combined with corticosteroids (at 1 mg/kg/d). Anakinra was started at 100 mg/d, with rapid efficacy on fever and cytopenia. However, efficacy decreased when anakinra was reduced to 100 mg/48h. The dose of anakinra had to be increased to 400 mg/d, combined with azacytidine. The inflammation only partially resolved and, at the patient's request, only comfort care was continued. The patient died one month later.

Patient 5:

A 52-year-old man was admitted to hospital with constitutional symptoms, persistent fever, and night sweats. HLH was diagnosed and a CT scan revealed enlarged supra- and subdiaphragmatic lymph nodes. Anakinra (100mg/d) was started, in combination with corticosteroids. The course was favourable with rapid resolution of fever and cytopenia. Lymph node biopsy identified Hodgkin's lymphoma and the patient was transferred to the haematology department to start chemotherapy.

Patient 6:

A 64-year-old man was diagnosed with an sHLH complicating a myelodysplastic syndrome (excess blasts secondary to radiochemotherapy for malignant thymoma). He had also feature of an associated neutrophilic dermatosis (Sweet's syndrome). VEXAS syndrome was ruled out. Due to the inefficacy of corticosteroids alone, anakinra was added, resulting in rapid control of biological inflammation. Azacytidine was started three months later and anakinra plus corticosteroids were rapidly weaned. The patient died four years after this episode, without any HLH recurrence.

Patient 7:

A 20-year-old man with a 17-year history of systemic-onset juvenile idiopathic arthritis (JIA) was admitted to hospital with fever and hepatitis, revealing sHLH. The patient had a long history of successive treatments for JIA (prednisone, MTX, CSA, AZA, anti-TNF, and anakinra, tocilizumab, and rituximab). Corticosteroids were started, but due to persistent fever after 48 hours, anakinra (200 mg/d) was added, resulting in rapid improvement of inflammatory and hepatic parameters. After seven days, the dose of anakinra was reduced to 100 mg/d. However, the onset of wrist arthritis and recurrence of biological inflammation required an increase in dose (to 200 mg/d), which was then continued long-term in combination with low-dose corticosteroids (7.5 mg/d).

Patient 8:

A 17-year-old woman was diagnosed with adult-onset Still's disease (AOSD) or AOSD-like disease (persistent fever, inflammatory arthralgias, sore throat, and biological inflammation), concurrent with primary infection with parvovirus B19. NK cell degranulation and perforin expression tests were normal. Corticosteroids were started. After three months, the disease relapsed (when corticosteroids were decreased beneath 15 mg/d), and the patient met the criteria for sHLH. Anakinra was started at 100 mg/d and arthralgia, fever and inflammation resolved in within the following 24 hours. Twenty days later, sHLH recurred, requiring high-dose i.v. methylprednisolone (1 g/d) combined with an increase in dose of anakinra (to 200 mg/d). CSA

was added after two additional months due to another relapse of AOSD (but without features of sHLH). Three months later, the patient had a new episode of sHLH, which was controlled by high-dose i.v. methylprednisolone and a new increase in dose of anakinra (to 200 mg/d). Recurrent episodes of urticarial rash after anakinra injection and a "feeling of swelling in the neck" led to a switch to MTX, which was not effective. Tocilizumab was subsequently started, leading to repeated allergic skin reactions. Finally, the patient responded well to canakimumab 150 mg/month, with good tolerance, allowing the corticosteroid to be reduced to 5 mg/d.

Patient 9:

A 41-year-old woman was admitted to hospital with a suspected haematological malignancy (lymphoma). She had persistent fever, constitutional symptoms, diffuse lymph node enlargement, and hepatosplenomegaly. Biological tests revealed increased acute phase reactants and features of sHLH. The left axillary and inguinal biopsies suggested a T-cell lymphoma, but clonality study did not show monoclonal lymphocyte proliferation; the bone marrow examination did not show any malignant infiltration. Corticosteroids allowed initial clinical and biological improvements. One month later, sHLH recurred and the patient received an infusion of etoposide (150mg/m^2), which was effective but only for a few days. Treatment with CSA (200 mg/d) was ineffective, and the patient had a major episode of sHLH with haemodynamic failure requiring a transfer to an ICU, an increase in corticosteroids (to 1 mg/kg/d), and a new infusion of etoposide (100 mg/m^2). The treatment resulted only in a partial improvement. She then had a skin rash and sore throat suggestive of AOSD. Anakinra was started (100 mg/d) and resulted in a dramatic efficacy. Five months later, the patient underwent a recurrence of sHLH, which was successfully treated by increasing the anakinra dose to 200 mg/d in combination with CSA. One year later, a recurrence of sHLH, was successfully managed by increasing anakinra alone to 200 mg/d. Anakinra (200 mg/d), combined with CSA and corticosteroids (5 mg/d) was then continued long-term without further attempts to reduce the dose. No evidence of lymphoma was found after a 4-year follow-up.

Patient 10:

A 61-year-old man was admitted to hospital with sHLH features associated with diarrhoea. The fever persisted under probabilistic ceftriaxone therapy. Anakinra (100 mg/d) was started to quell cytokine storm, resulting in fever resolution within 48 hours. The serological work-up finally revealed a *Rickettsia typhi* infection (titre 1024UI/l) and a treatment with doxycycline allowed the rapid discontinuation of anakinra. The outcome was favourable, without any recurrence of sHLH after a one-year follow-up.

Patient 11:

A 45-year-old woman was diagnosed with AOSD complicated by sHLH. The course was initially favourable on prednisone (1 mg/kg/d) and anakinra (200mg/d), but marked by injection-site pain. Anakinra had to stopped after 14 days and switched to tocilizumab (due to steroid dependency at 10 mg/d of prednisone) with good efficacy. Unfortunately, the patient developed abdominal dermohypodermitis and skin intolerance to subcutaneous tocilizumab injections. Treatment was switched to MTX (20 mg/week) was carried out, allowing corticosteroid weaning.

Patient 12:

A 51-year-old man underwent emergency liver transplantation for portal hypertension and uncontrolled bleeding in the context of NASH cirrhosis. The post-transplant period was complicated by multi-visceral failure, associated with portal thrombosis and major icteric cholestasis, with no infectious aetiology found despite a broad workup. Liver biopsy revealed a discrete lymphocytic infiltrate with major cholestatic lesions. Clinical and biological findings were consistent with sHLH, although some criteria appeared to be secondary to the major cholestasis. High-dose i.v. methylprednisolone (500 mg/d) was administered due to suspicion of atypical graft rejection or inflammation of undetermined origin. Because of only partial efficacy, anakinra (100 mg/d) was added to corticosteroids, which controlled inflammation and improved liver function. After 14 days, anakinra was reduced to 100 mg/48h and then stopped after 21 days because the patient developed dermohypodermitis. Corticosteroids were continued alone and weaned after four months.

Patient 13:

A 35-year-old woman presented with persistent fever, constitutional symptoms, and cholestatic hepatitis. The patient met the criteria for sHLH. Anakinra (200 mg/d) was started as monotherapy with good efficacy. Liver biopsy finally eventually revealed granulomatous hepatitis with non-caseous necrosis and direct examination confirmed disseminated tuberculosis. Anti-tuberculosis treatment was started and anakinra was switch to oral corticosteroids (to simplify drug administration), with a decreasing dosage, weaned after the tuberculosis treatment was stopped at six months.

Patient 14:

A 28-year-old woman was admitted to hospital with persistent fever revealing disseminated tuberculosis with lung, lymph node, and spleen involvement. She had undergone renal transplantation three years previously, due to granulomatous tubulointerstitial nephritis, labelled as sarcoidosis. Anti-tuberculosis therapy was started, but the patient presented an IRIS, with sHLH criteria. High-dose i.v. methylprednisolone was ineffective. Anakinra (200 mg/d) was started with dramatic efficacy. Anakinra was continued as monotherapy during the first months of anti-tuberculosis treatment, because the patient had recurrent episodes of fever at each attempt of withdrawal. Anakinra was definitively stopped after four months.

Patient 15:

A 6-year-old boy presented with persistent high fever associated with an urticarial rash and pain in all extremities, and clinic-biological criteria of sHLH. There was rapid clinical deterioration and haemodynamic failure secondary to cardiac tamponade, which was drained in paediatric intensive care. A course of IVIG (1 g/kg) was administered due to suspicion of Kawasaki disease. Etiological investigations were negative except for a positive parvovirus PCR. In the absence of improvement, corticosteroids (2 mg/kg/d) were started but were ineffective. Because JIA was further suspected, anakinra (2 mg/kg/d) was started but resulted in no improvement within the first three days. High-dose i.v. methylprednisolone (20 mg/kg/d) was administered for 3 days, combined with CSA (3 mg/kg/d) and a new course of IVIG, resulting in a satisfactory control of the inflammatory parameters. The final diagnosis of JIA was retained and anakinra was resumed as background treatment. CSA was stopped one month later, then corticosteroids at four months, and finally anakinra at six months. Two years later, the patient had an JIA relapse (without sHLH criteria), which was successfully treated by anakinra (100 mg/d).

Patient 16:

A 4-year-old girl was admitted to hospital for the management of sHLH. She had been diagnosed a month earlier as having JIA in the setting of persistent fever with a fleeting rash, with no initial criteria for sHLH. Her treatment consisted of prednisone and anakinra (2.5 mg/kg/d). sHLH was treated by increased dose of corticosteroids (4 mg/kg), combined with increased doses of anakinra (5 mg/kg/d), with good efficacy. Canakinumab (75 mg/3 weeks) was started two months later, with initial efficacy but a secondary relapse of JIA (without sHLH criteria) at six months. Canakinumab was switched to tocilizumab. The patient underwent a severe allergic reaction on the third injection of tocilizumab, leading to a definitive contraindication. Treatment with baricitinib was then started with good efficacy, allowing the weaning of corticosteroids.

Patient 17:

A 55-year-old woman presented with a three months history of constitutional symptoms, with weight loss (30 kg), associated with fever (40°C) and night sweats. Investigations revealed the criteria for sHLH diagnosis. Anakinra (200 mg/d) was started during the etiological work-up, allowing a rapid resolution of fever and inflammatory markers. The PET scan was suggestive of hematologic malignancy with several hypermetabolic lymph nodes. Bone marrow and lymph node biopsies led to the diagnosis of Hodgkin's lymphoma. The patient was transferred to haematology and anakinra was stopped at the initiation of chemotherapy.

Patient 18:

A 58-year-old man was admitted to hospital with *Streptococcus suis* bacteriemia complicated by arthritis of the right shoulder. He received i.v. amoxicillin and gentamicin, followed by oral rifampicin and levofloxacin. He was re-admitted to hospital one month later because he had a septic shock with sHLH criteria despite ongoing oral antibiotic therapy. He received vasopressor support, tazocillin/tazobactam and i.v. methylprednisolone (1 mg/kg/d). Due to incomplete efficacy, anakinra was added 24 hours later, resulting in a favourable outcome of the sHLH and a rapid weaning of corticosteroids. Anakinra was stopped after 17 days. The etiological work-up did not find any other cause than a post-infectious inflammatory process.

Patient 19:

A 35-year-old woman presented with fever, dyspnoea and sore throat. The cervical CT scan revealed a parapharyngeal abscess. The course was rapidly marked by a cardiogenic shock with troponin elevation (1000 ng/L), leading to the diagnosis of myocarditis. Extensive antibiotic therapy and vasopressor support were combined with cardioprotection. Because of persistent fever and clinic-biological criteria of sHLH, anakinra was started. The autoimmune work-up was negative and the cardiac MRI was suggestive of a stress cardiopathy. The outcome was favourable under antibiotic therapy and anakinra, which was weaned at D8. A control of the cervical CT scan revealed only a regressing lymphadenopathy, which could not be biopsied. The final diagnosis of *takotsubo* with sHLH in the setting an ENT infection was retained.

Patient 20:

A 58-year-old man presented with septic shock and sHLH criteria, complicating a knee prosthesis infection by *Staphylococcus aureus* and *Candida krusei*. He received a combination of anti-microbial chemotherapy and IVIG, without efficacy. Corticosteroids did not bring any

improvement. Anakinra was started as a third-line treatment for sHLH, with a dramatic efficacy. The course was slowly favourable and the knee prosthesis was removed. Anakinra was stopped at 14 days, with no further recurrence of sHLH.

Patient 21:

A 77-year-old man was admitted to hospital with multiple intra-abdominal abscesses and a purulent effusion in the left iliac fossa. He had a history of abdominal aortic stenting, a BCGitis (bladder cancer), and a Bricker surgery three months prior to the current presentation. *Candida albicans* infection was identified within the effusion and he received antifungal treatment with caspofungin. However, fever persisted and cytopenia developed, leading to the diagnosis of sHLH. At the same time, a major carcinomatous recurrence with liver tumoral infiltration was diagnosed. Treatment with anakinra (100 mg/d) was started to control the cytokine storm. Within 48h, fever resolved and the inflammatory parameters decreased, allowing the reduction of anakinra dose to 100 mg/48h after seven days. Anakinra was stopped after 14 days, with no further recurrence of sHLH.
